# Investigation on the Temperature Control Accuracy of a Print Head for Extrusion 3D Printing and Its Improved Design

**DOI:** 10.3390/biomedicines10061233

**Published:** 2022-05-25

**Authors:** Peng Zhang, Qiang Gao, Kaicheng Yu, Yifeng Yao, Lihua Lu

**Affiliations:** 1 School of Mechatronics Engineering, Harbin Institute of Technology, Harbin 150001, China; zp@hit.edu.cn (P.Z.); 20s108274@stu.hit.edu.cn (K.Y.); 21s108310@stu.hit.edu.cn (Y.Y.); 2 Chongqing Research Institute of HIT, Chongqing 400000, China; 3 School of Astronautics, Harbin Institute of Technology, Harbin 150001, China

**Keywords:** temperature control accuracy, rheological property, extrusion 3D printing, printing temperature, filament diameter

## Abstract

For the extrusion 3D printing process, the printing temperature has a significant impact on the filament formation process because the rheological properties of the printed materials are extremely thermal sensitive, which requires a high temperature control accuracy of the print head. This paper presents a numerical and experimental investigation on the temperature field of a homemade print head. A finite element simulation model for analyzing the temperature field of the print head was established, by which the temperature distribution inside the print head can be acquired. Moreover, to improve the temperature control accuracy, an improved configuration was proposed, and two schemes were compared. The temperature control error dropped from 28% to 6.2% with the improved print head, which was verified experimentally. Furthermore, printing trials were conducted by the optimized print head. The filament diameter could be regulated by changing the temperature of the print head, which validates the feasibility to control the filament diameter during the extrusion process via temperature regulation.

## 1. Introduction

As a promising method to manufacture in vitro tissue and disease models in biofabrication, three-dimensional (3D) printing enables the fabrication of various complex structures with varying biocompatible materials [1], which manifests great potential in the fields of tissue engineering and regenerative medicine [2,3].

Currently, accessible methods for 3D printing technologies in biofabrication mainly include extrusion printing [4,5,6], photopolymerization-based printing [7,8,9], and inkjet-based printing [10,11,12]. Among these 3D printing techniques, extrusion 3D printing is the most widely adopted method due to its low cost and great availability [13]. It is compatible with a wide range of materials with various rheological properties, and the fabrication process with an extrusion 3D print device is convenient to conduct. The extrusion process is typically actuated by an extruding plunger or compressed air to extrude the materials from a cartridge through a tiny nozzle. Filaments with constant diameters can be produced and then printed with a predefined path onto a two-axis linear stage. Ultimately, a complex 3D structure can be fabricated by a layer-by-layer deposition process.

A wealth of research has been conducted in biofabrication based on extrusion 3D printing. Miller et al. [14] printed a structure with channels based on extrusion 3D printing and hydrogel casting, and carbohydrate glass was utilized as a sacrificial material. It demonstrated that the fabricated constructs have an excellent ability to sustain the metabolic function of primary rat hepatocytes. Merceron et al. [15] fabricated a construct involving a complex muscle–tendon interface by integrating the structure of the muscle phase and tendon phase by extrusion 3D printing. The printing process was conducted with multiple print heads, which can print cell-laden hydrogels and biodegradable polymers. Jakab et al. [16] developed vessel-like tissue constructs based on extrusion 3D printing to mimic blood vessels. The cell-laden units and supportive units were printed together first, and then the supportive units were removed to obtain the constructs with predefined shapes. Hinton et al. [17] prepared a complex biological structure inside the suspended hydrogels based on extrusion 3D printing. Models of the human femur and arterial tree were obtained through 3D computed tomography imaging and fabrication in their research. Lee et al. [18] adopted the collagen ink and a high-concentration cell ink to fabricate the components of the human heart. These structures were able to achieve some functions of the human heart.

The vast majority of the bioink materials utilized in extrusion 3D printing are polymer solutions with complex rheological properties. They generally show excellent mechanical properties and biocompatibility at room temperature, which make them ideal candidates to fabricate scaffolds to provide structural support for cell proliferation [19]. However, their viscosity and viscoelasticity, which has a significant impact on the filament formation process, are usually sensitive to temperature variation [20]. Some materials, such as the mixture of polycaprolactone (PCL) and β-tricalcium phosphate (β-TCP), the mixture of PCL and Zirconium dioxide (${\mathrm{ZrO}}_{2}$), and polylactide-co-caprolactone (PLCL), can only be printed after a heating procedure. An improper printing temperature may cause filament wrinkle or even fracture. Therefore, the temperature is a significant process parameter of the extrusion 3D printing process, which should be controlled accurately. Park et al. [21] mixed the PCL and β-TCP into blends to manufacture a complex scaffold with extrusion 3D printing. The blends were heated and printed at 130 °C to ensure the continuous filament formation during the printing process. The printed scaffold shows good bioactivity by a cell differentiation experiment of mouse mesenchymal stem cells. Chang et al. [22] prepared a novel tissue engineering functional skin based on extrusion 3D printing. During the fabrication of PLCL, the temperature was controlled at 150 °C. The printed PLCL scaffold carrying minimal functional units of skin and collagen gel has increased the healing speed of wounds and has shown great potential in full-thickness skin wound healing. Kang et al. [23] adopted an extrusion 3D printer with a heating unit to provide the desired temperature for the manufacturing process of a PLCL thick tissue with many interconnective pores. The constructs were fabricated with cell-laden hydrogel along with pluronic hydrogel or PCL and have shown robust tissue formation, such as bone and cartilage, after being implanted. Wang et al. [24] fabricated groups of biocompatible scaffolds with the blend of PCL and ${\mathrm{ZrO}}_{2}$ at 105 °C. The water absorption capacity, mechanical properties, and bioactivity of these printed structures were investigated.

Although a consensus has been reached that temperature has a dramatic impact on the extrusion process of the material, the temperature control accuracy of the printed material during the 3D printing process is seldom investigated. The typical dimensions of the printing nozzle are in the range of several hundred micrometers, which is much small than the size of the temperature sensor and heat source. Hence, installing the heat source and temperature sensor around the nozzle is impractical [25]. During the working process of the print head, the heat generated by the heat source transfers inside the print head via thermal conduction. Besides, the convective heat transfer between the external surface of the print head and the surrounding air takes heat to the ambient environment. The temperature distribution inside the print heat is uneven when the thermal balance is finally reached. It indicates that the temperature near the nozzle may deviate from the observed temperature of the sensor. When the temperature of the printing material inside the tiny nozzle is below its melting temperature, the polymer will be solidified [26]. Once the nozzle is blocked by the cooled material, the extrusion process will be interrupted immediately, which consequently leads to a waste of time and materials. Therefore, the temperature of the material inside the nozzle is the most critical to be controlled in the extrusion 3D printing process rather than the temperature recorded by the sensor.

In this research, the temperature field of a homemade print head was investigated numerically and experimentally. Thermal analysis of the print head based on the finite element method (FEM) was conducted to analyze its temperature distribution. Besides, an improved configuration with two schemes was proposed and compared for improving the temperature control accuracy of the nozzle. Moreover, a temperature-measuring experiment was conducted to analyze the temperature control accuracies of the initial and optimized 3D print heads. Eventually, groups of 3D printing trials for PLCL were conducted by the optimized print head with various temperatures. The diameters of printed filaments can be regulated by changing the temperature of the optimized 3D print head.

## 2. Configuration of an Extrusion 3D Print Device and Its Improved Design

### 2.1. The Initial Configuration of an Extrusion 3D Print Device

**Figure 1:**
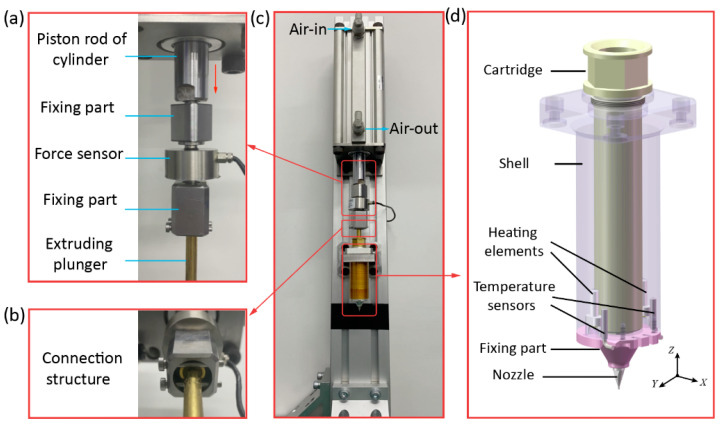
The configuration of a self-designed extrusion 3D printing device. (**a**) The enlarged view of the force sensor; (**b**) The connection structure; (**c**) The self-designed 3D print device; (**d**) The homemade print head.

Figure 1 demonstrates a self-designed 3D printing device. It mainly consists of an air cylinder (CDQ2A63-150DCZ, SMC, Tokyo, Japan), a force sensor (ZNLBM-500KG, Bengbu Zhongnuo Electronic Technology Co., Ltd., Bengbu, China), and a homemade print head. The piston rod of the cylinder, the force sensor, and the extruding plunger of the print head were connected in series as shown in Figure 1a–c. Since the polymer melt generally has a high viscosity, the pressure of the air source provided by the air compressor is commonly lower than 0.8 MPa, which may be incapable to provide adequate extrusion force. The air cylinder can amplify the extrusion force provided by compressed air, which therefore can provide a larger extrusion force. Hence, a cylinder was adopted to actuate the print head in the self-designed 3D printing device. The force sensor was employed to observe how the extrusion force acted on the material in the printing process.

Figure 1d details the configuration of the homemade print head. It comprises a cartridge, a shell, two heating elements, two temperature sensors, a fixing part, and a nozzle. To control the temperature of the materials inside the 3D print head, two metal-ceramic heaters were adopted as the heating elements, and two temperature sensors (Pt1000) were installed near the heating elements to observe their temperature. The heating elements and temperature sensors were both fixed on the shell tightly with the fixing part. To promote the heat transfer efficiency, the shell is made of the aluminum alloy due to its high thermal conductivity. The cartridge is made of food-grade (304 grade) stainless steel to accommodate the bioink material. During the 3D printing process, the heat can be transferred from the heating elements to the printed material through the shell and the cartridge.

To accurately control the temperature of the materials around the nozzle, the temperature sensors should be installed as close to the nozzle as possible. However, the diameter of the nozzle adopted is usually less than 1 mm. Considering the structural dimension of the temperature sensor, installing the temperature sensors around the nozzle is impractical. Thus, installing the heating elements at the bottom of the shell is a compromised choice in the current design, and the temperature recorded by the temperature sensor was regarded as the temperature at the nozzle approximately.

### 2.2. The Improved Configuration of the Print Head

On account of the convective heat transfer between the surrounding air and the heated print head, the heat loss through the external surface of the print head could produce a thermal gradient in the temperature field of the print head. This phenomenon is especially obvious around the nozzle area because its volume is much smaller than the shell and the cartridge. It will cause a large deviation between the target temperature and the real temperature at the nozzle, which decreases the temperature control accuracy of the filament formation process.

**Figure 2:**
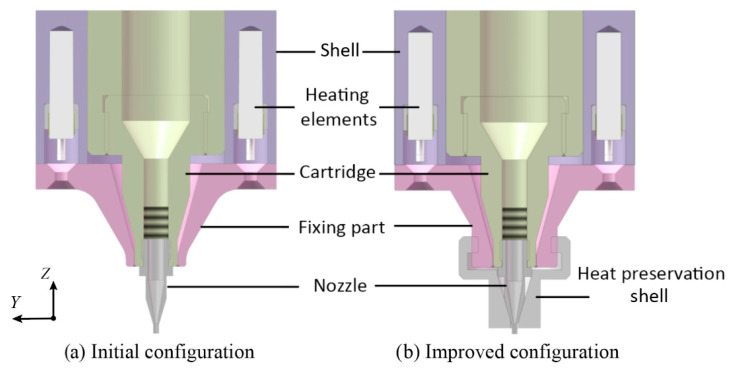
The initial and improved configurations of the homemade print head.

To improve the temperature control accuracy, the temperature dropping at the nozzle should be alleviated. An improved configuration based on the initial print head was proposed in this section, as shown in Figure 2. The basic idea of the improved configuration is to restrict the heat loss from the external surfaces of the nozzle. Therefore, a heat preservation shell was designed to cover the nozzle entirely. There are two schemes for the material design of the heat preservation shell. The first scheme is manufacturing the heat preservation shell with polyetheretherketone (PEEK). The heat convection between the nozzle and the air is substituted by the heat conduction between the nozzle and the PEEK heat preservation shell. The PEEK can be utilized to prevent heat loss due to its poor thermal conductivity, which means the temperature drop of the nozzle could be declined by this scheme. The second scheme is to manufacture the heat preservation shell with aluminum alloy. Unlike PEEK, the aluminum alloy shows excellent thermal conduct capability to transfer the heat from the heating elements to the nozzle through the metal shell and fixing part. It can provide additional heat to the nozzle in comparison to the initial configuration of the print head. The above proposed two schemes both show validity in principle. In the following sections, the temperature control accuracy of the initial print head and its improved configuration will be investigated and compared numerically to decide the material selection.

## 3. Thermal Analysis of the Print Head

### 3.1. FEM Modeling for the Print Head

**Figure 3:**
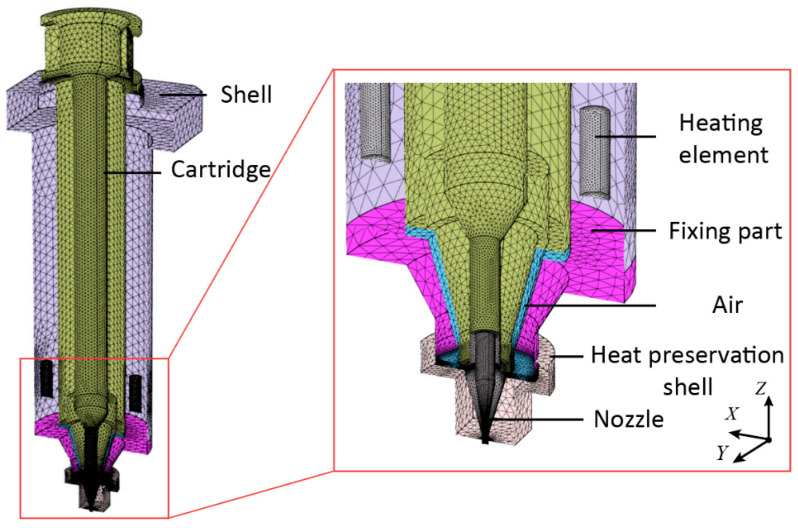
The computational mesh of the print head.

To accurately estimate the temperature distribution of the print head in its working condition, the steady-state thermal simulation model of the print head is established based on FEM. The thermal boundary conditions of the print head are analyzed, and the computational mesh of the print head is shown in Figure 3. In order to reduce the difficulty of the modeling process and improve the calculation efficiency, the geometrical features, such as the chamfers and screw holes, were ignored to simplify the FEM model. The heating element is regarded as a temperature boundary with a constant temperature. The external surfaces of the print head are simplified as the convective heat transfer boundary. Besides, to enhance the simulation accuracy, the thermal contact resistance of the contact surfaces between two adjacent parts is considered. Furthermore, the internal air at the gap between the cartridge and the fixing part was also taken into consideration as shown in the enlarged view of the Figure 3. The total mesh number in the FEM model reached 1,400,000. The FEM modeling process of this paper can be regarded as a case study of the modeling approach for simulating the heat source, convection heat transfer coefficients, and thermal contact resistance. It can also be adopted for the thermal analysis of other print heads.

### 3.2. The Boundary Conditions of the Simulation Model

#### 3.2.1. Heat Source of the Print Head

In the designed print heat, the heating elements were installed into the shell as the heat source of the print head. Because the temperature sensors are close to the heating elements, the temperature deviation between the heating element and the sensor is ignorable. Therefore, the heating elements are considered as volumes with a constant temperature in the FEM model. In the simulations, the temperature of the heating elements was defined to several target temperatures of 60, 80, 100, 120, 140, 160, and 180 °C.

#### 3.2.2. Convection Heat Transfer Coefficients

In order to obtain the heat transfer coefficient, the types of heat transfer regime should be determined first. Two dimensionless numbers, the Reynolds number ($R_{e}$) and the Grashof number ($G_{r_{L}}$), are utilized to determine the type of heat convections [27]:

(1) $$G_{r_{L}}=\frac{g\beta \left(T_{s}-T_{\infty }\right)L^{3}}{v^{2}}$$

(2) $$R_{e}=\frac{\rho uL}{v}$$

where g is the gravitational acceleration and β is the volumetric thermal expansion coefficient. $T_{s}$ and $T_{\infty }$ are the temperature of the surface and surrounding air, respectively. L is the characteristic length, and v is the kinematic viscosity of the air. Due to the calculated $\frac{G_{r_{L}}}{R_{e}{}^{2}}$ is much larger than 1, the heat transfer regime between the heat head and air was considered as the natural convection. For the natural convection:

(3) $$h=\frac{0.59k}{L}G_{r_{L}}P_{r}$$

where $P_{r}$ is the Prandtl number, which is a physical property of air related to the temperature, and k is the thermal conductivity of air.

#### 3.2.3. Thermal Contact Resistance

The thermal contact resistance occurs at the contact surfaces of two different parts due to the irregularity of the two surfaces. This causes a much lower thermal conductivity at the contact surface of two parts than that inside a single part. In the research, the Cooper–Mikic–Yovanovich contact theory is utilized [28] to figure out the thermal contact resistance between each contact surface in the simulation model:

(4) $$h_{c}=1.25k\frac{m_{asp}}{\sigma_{asp}}{\left(\frac{p}{H_{c}}\right)}^{0.95}$$

where $h_{c}$ is the contact conductance; $m_{asp}$ is the number of contacts in a given area; $\sigma_{asp}$ is the standard deviation of profile heights, which relates to the processing method; p is the pressure acting on the surface; and $H_{c}$ is the microhardness of the contact surface, which is material related.

## 4. Analysis and Experimental Verification for the Simulation Results

### 4.1. Analysis of the Simulation Results

**Figure 4:**
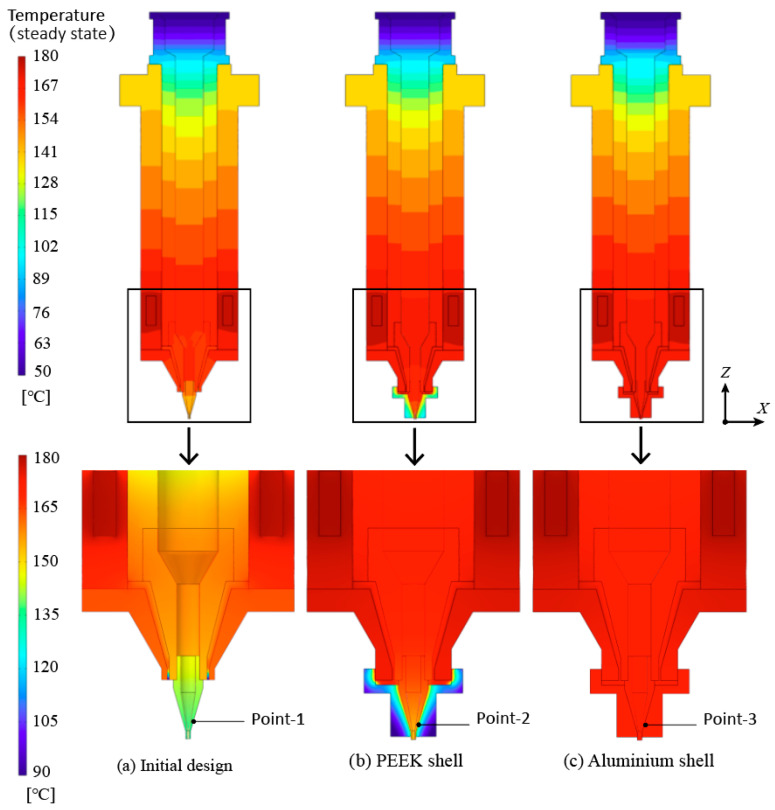
Temperature distributions of the print heads with heat source temperature of 180 °C.

Figure 4 illustrates the temperature distributions of the initial and the improved print heads acquired with a heat source temperature of 180 °C. In the overall view of Figure 4a, it can be seen that the maximum temperature is 180 °C around the heating element, while the minimum temperature occurs at the top of the cartridge, which is about 60 °C. The temperature drop at the top of the cartridge is attributed to its large distance to the heat source. This phenomenon can also be observed in the improved print heads as shown in Figure 4b,c. Despite this severe temperature drop, it has little effect on the filament formation process. Because the printed material inside the cartridge was extruded from the top to the bottom during the extrusion process. The low-temperature materials at the top of the cartridge will be heated again when they pass by the high-temperature area around the heat source. Therefore, the temperature drop at the top of the cartridge is of no account for the temperature control accuracy of the print head.

According to the enlarged view of Figure 4a, an obvious temperature decrease can also be found in the area near the nozzle. It mainly results from the convective heat transfer taking heat into the surrounding atmosphere. During the printing process, the temperature of the materials above the nozzle declines gradually when these materials are extruded toward the nozzle. The temperature of the materials inside the nozzle has a direct impact on the filament formation process. It proves that the temperature of the nozzle should be considered as the target temperature of the temperature control system instead of the temperature of the heating source.

The enlarged views of the Figure 4b,c present the temperature contour of the improved configuration with the heat preservation shell, which indicates that the heat preservation shell has a dramatic impact on the temperature of the nozzle area. The nozzle temperature improves obviously by the heat preservation shell, and the aluminum heat preservation shell shows higher heat preservation capability.

**Figure 5:**
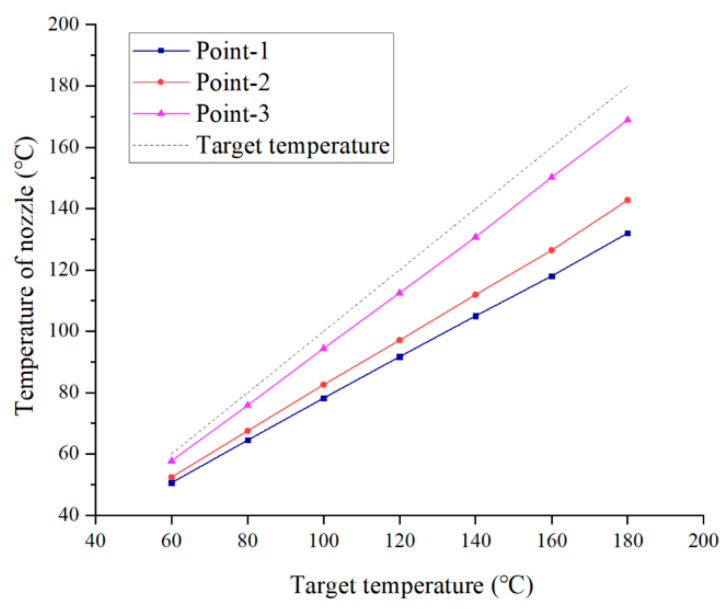
Steady-state temperatures with varying target temperatures.

In order to comprehensively compare the temperature preservation ability of the two preservation shells and the temperature control accuracy in varying working conditions, Figure 5 presents the temperature curves of three points under different heat source temperatures. As shown in Figure 4, point 1 is located at the surface of the nozzle for the initial design, while point 2 and point 3 are located at the interfaces between the nozzles and the heat preservation shell. It shows that the nozzle temperature prevented by the aluminum shell is closer to the desired temperature. Hence, the second scheme that adopts the aluminum alloy heat preservation shell has higher temperature control accuracy.

### 4.2. Experimental Verification for the Simulation Results

**Figure 6:**
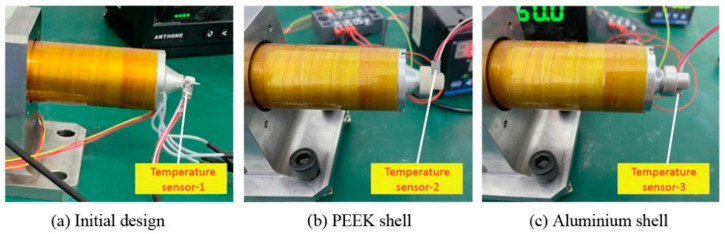
Experimental setup for the measurement of temperature.

To verify the calculation accuracy of the FEM model and the accurate temperature control ability of the improved printing head, groups of experiments were conducted to measure the actual nozzle temperature with different target temperatures. As demonstrated in Figure 6a–c, three temperature sensors were adopted to measure the nozzle temperature for the initial and improved print heads. Besides, in order to accurately measure the temperature at the surfaces of nozzles that were covered by the heat preservation shells, a tiny hole was drilled on the heat preservation shells, so that the temperature sensors could be installed into the shells to contact the nozzle surfaces.

**Figure 7:**
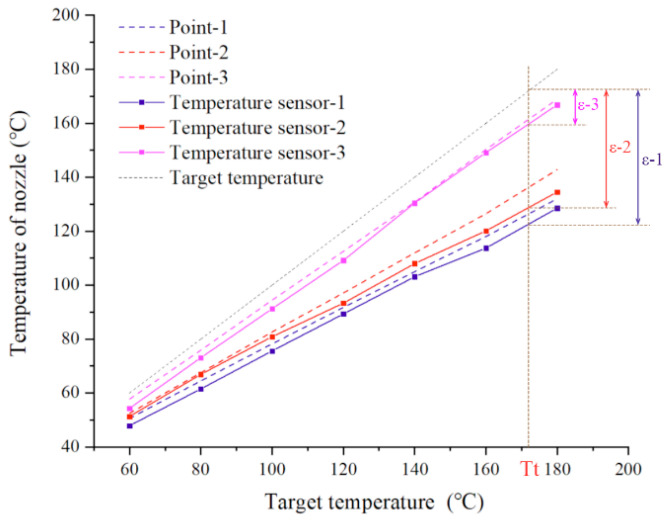
Measurement of temperature at the nozzle.

Figure 7 compares the measured temperature curves with the sensors and the simulation results. It indicates that the overall trend of the measured curves agrees well with that of the simulated curves, which verifies the accuracy of the simulation model. To further evaluate the temperature control accuracy of the initial and improved print heads, a black dashed line of the target temperature curve was also demonstrated in Figure 7. The error (ε) between the target temperature and the measured temperature of the nozzle at a certain temperature (Tt) is defined as shown in Figure 7. It can be seen that the temperature error of the improved configuration with aluminum shell (ε-3) is much lower compared with the initial configuration (ε-2) and the configuration with the PEEK shell (ε-1). When there was no protection for the nozzle, the maximum error of temperature control reached 28%. The maximum error of the temperature control of configuration with the PEEK shell declined to 21.1%. By contrast, with the heat preservation shell made of aluminum alloy, the maximum error of the temperature control dropped to 6.2%. This manifested that the improved print head is feasible to enhance the temperature control accuracy, and the aluminum shell has a better heat preservation ability than the PEEK shell.

## 5. Extrusion 3D Printing Experiment for PLCL

During the filament formation process of the extrusion printing, the rheological property of the printed materials has a significant impact on the filament diameter due to the extruding swell phenomenon, as the rheological properties of a certain material are sensitive to its temperature. The diameter of the printed filament may be regulated by accurately changing the printing temperature. To validate its feasibility, extrusion printing trails were implemented with the above improved print head as described in this section.

### 5.1. The Rheological Investigation of PLCL

The PLCL is a frequently adopted material in scaffold manufacturing because of its good bioactivity and biocompatibility. Therefore, it is adopted to conduct the extrusion 3D printing trials. The PLCL is a kind of polymer blends of poly(L-lactide) (PLLA) and PCL. The formulation of PLCL adopted in this study was 50:50, which shows high efficiency to transfer from a rubbery state to a viscous state above melting temperature [29,30]. The raw material of PLCL was purchased from the Jinan Daigang Biomaterial Co., Ltd., Jinan, China. To validate the temperature sensitivity of its rheological properties, the rheological investigation was conducted first on a HAAKE MARS III rotation rheometer (Thermo Fisher Scientific, Shanghai, China). The oscillatory temperature sweep mode was adopted to test the dynamic complex modulus and viscosity.

**Figure 8:**
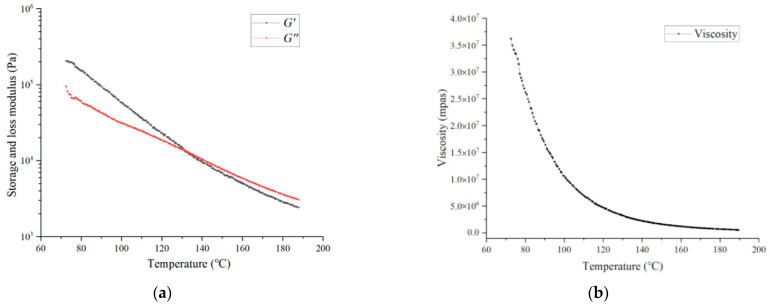
(**a**) Storage and loss modulus of PLCL with various temperatures; (**b**) viscosity curves of PLCL with various temperatures.

Figure 8 presents the storage, loss modulus, and viscosity of PLCL with varying temperatures. Figure 8a demonstrates that both the storage modulus (G’) and the loss modulus (G”) declined gradually as the temperature was increasing. The storage modulus of PLCL was much higher than the loss modulus at low temperature, while their difference got smaller with increasing temperature. This means the elasticity of PLCL melt played a dominant role in this condition, which consequently resulted in a poor fluidity of the melts. When the temperature reached 130 °C, a cross point of these two curves appeared. Then, the loss modulus was higher than the storage modulus after this point. It indicates that the fluidity of PLCL melts gets stronger with further temperature incensement when the temperature is higher than 130 °C. It implies the PLCL will be easier to be extruded due to the enhanced fluidity at this condition. Figure 8b shows the viscosity curve of PLCL melts. It shows that the viscosity of PLCL melts has a sharp decrease with the growth of temperature at first. As the temperature keeps going up, the decline rate of the viscosity of PLCL gradually becomes slow. This also illustrates that the temperature growth could enhance the fluidity of PLCL melts.

### 5.2. The Influence of Temperature on the Diameter of Printed Filaments

**Figure 9:**
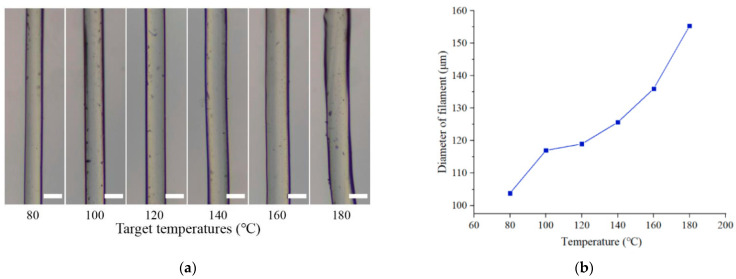
(**a**) Micrographs of printed PLCL filaments with varying temperatures, (**b**) diameters of printed filaments. Scale bar: 100 μm.

Before the extrusion 3D printing, the PLCL was put into the cartridge first and heated at a certain temperature for 1 h. Six temperatures of 80, 100, 120, 140, 160, and 180 °C were selected as the target temperatures for the extrusion 3D printing trials. A 100 μm nozzle was adopted for the printing process. In each group of experiments, the extrusion force was set to a constant value of 40 kg. Then, the PLCL filaments were extruded under the action of the extruding plunger. The printing experiments at a certain temperature were conducted three times, and the printed filaments were collected and observed by an optical microscope (PH100, Phenix, Shangrao, China). To measure the size of printed filaments, the diameter of each filament was calculated by comparing the images with the scale bar. For each filament, diameters at three points were selected and the average value of them was considered as the diameter of the filament. The micrographs of PLCL filaments printed in six temperatures were shown in Figure 9a, and the average values of the calculated filaments were demonstrated in Figure 9b. It can be seen that the diameters of printed filaments present an upward trend with the increase in controlled temperature.

The relationship between the temperature and the diameters of printed filaments can be explained as follows. Due to the extruding swell phenomenon, the diameter of extruded filament is generally larger than the nozzle diameter. The reason of the extruding swell phenomenon of polymers is rather complex compared with that of the Newtonian fluid [31]. According to a widely accepted theory of polymer conformation, the extruding swell phenomenon was highly related to the entropic elasticity during the printing process of PLCL [32]. Before the PLCL melts, entering the nozzle, each PLCL molecule maintains its polymer conformation of a random coil. These molecules are equipped with maximum conformational entropy. Then, as the melts enter the nozzle, the orientation of PLCL molecular chains changes under the velocity gradient of PLCL flow. This consequently lowers the conformation number of the PLCL molecules, which leads to the decrease of conformational entropy. Due to the Brownian motion of the molecules, the physical entanglements caused by the orientation of PLCL molecular chains relax in the nozzle, and the decreased entropy recovers in the meantime. The longer it spends in the nozzle, the more entropy can be recovered. After the PLCL leaves the nozzle, the orientation of molecules resulting from the velocity gradient finally disappears and the left physical entanglement of polymer chains relaxes. The conformation of each PLCL molecule gets back to the random coil, which possesses maximum conformational entropy. This leads to the extruding swell phenomenon that shows that the diameters of printed filaments were always larger than the diameter of the nozzle. However, under the effect of Brownian motion of the molecules, the longer it takes in the nozzle, the weaker the extruding swell phenomenon would be. Because the flow of PLCL melts inside the nozzle can be regarded as a Poiseuille flow [33], the decrease of viscosity resulting from the growth of temperature would lead to an improvement of the flow velocity and a decline of the time spent inside the nozzle. This means the increase in temperature would ultimately lead to the growth of printed filament diameters.

This phenomenon demonstrated that it is feasible to control the diameter of printed filament via the accurate control of temperature. The improved print head has potential application for the 3D printing devices when the filament diameter regulation is necessary.

### 5.3. The Filaments Printed by the Initial and Improved Print Heads

**Figure 10:**
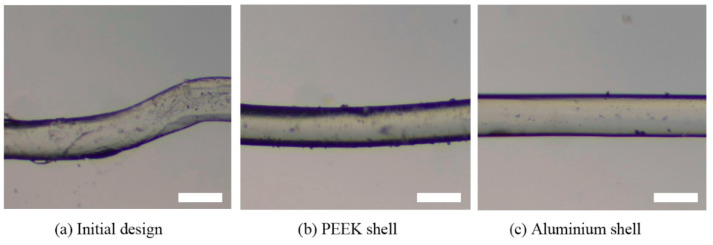
Print samples at the target temperature of 100 °C. Scale bar: 100 μm.

The print heads with the PEEK shell, aluminum shell, and the initial design were employed to extrude the PLCL filaments at the target temperature of 100 °C. The micrographs of the printed filaments are presented in Figure 10. It can be seen that the shape of filament printed with the initial print head is not as stable as that fabricated with the improved print heads, and the PLCL filament extruded by the print head with aluminum shell shows the smoothest surface and best shape. The reason of this phenomenon derives from that the actual temperature at the nozzle will be lower than the target temperature without the protection of heat preservation shell. As the viscosity of PLCL increases sharply with the decline of the temperature, the rheological properties of PLCL melts inside the nozzle are not in a suitable condition for filament formation process, which therefore leads to a bad printing result of PLCL filaments. While the print head with the aluminum shell has the best temperature control accuracy, the temperature of the PLCL materials inside the nozzle is closer to the target temperature, which consequently shows better print result. This phenomenon demonstrates the significance of high accuracy temperature control during the 3D printing process.

## 6. Conclusions

This research investigates the temperature control accuracy of a homemade extrusion print head. An improved configuration is proposed and verified experimentally. Printing trials were also conducted to change the filament diameter by regulating the printing temperature. The main conclusions of this paper are as follows:

(1) An improved configuration for a self-designed 3D print head was proposed to enhance the temperature control accuracy of the nozzle.
(2) A thermal analysis model of the print head system was established based on FEM, and the temperature contour of the print head was acquired, which proves that the nozzle temperature should be regarded as the target temperature of the temperature control system.
(3) The temperature control error declined from 28% to 6.2% with the improved print head with an aluminum heat preservation shell, which was validated numerically and experimentally.
(4) A group of print trials with PLCL was conducted, which verifies the feasibility of regulating the filament diameter by accurately controlling the temperature of the nozzle.

## Data Availability

Not applicable.
